# Supplementary material for: Effects and Impacts of Different Oxidative Digestion Treatments on Virgin and Aged Microplastic Particles
Source: Polymers (Basel). 2022 May 11;14(10):1958. doi: 10.3390/polym14101958 (PMC9146394; doi:10.3390/polym14101958)
Supplement: Supplementary file 1 [file polymers-14-01958-s001.zip › polymers-1686831-supplementary.pdf]

Supplementary material

Effects and impacts of different oxidative digestion treatments on virgin and aged Microplastic particles

Ilaria Savino [1,2], Claudia Campanale [1*], Pasquale Trotti [3], Carmine Massarelli [1], Giuseppe Corriero [2] and Vito Felice Uricchio [1]

[1] Italian National Council of Research - Water Research Institute – Bari, Italy
[2] Department of Biology, University of Bari Aldo Moro, Bari, Italy
[3] Dipartimento di Scienze del Suolo, della Pianta e degli Alimenti – Sezione di Entomologia e Zoologia Agraria - University of Bari Aldo Moro, Bari, Italy
* Correspondence: claudia.campanale@ba.irsa.cnr.it

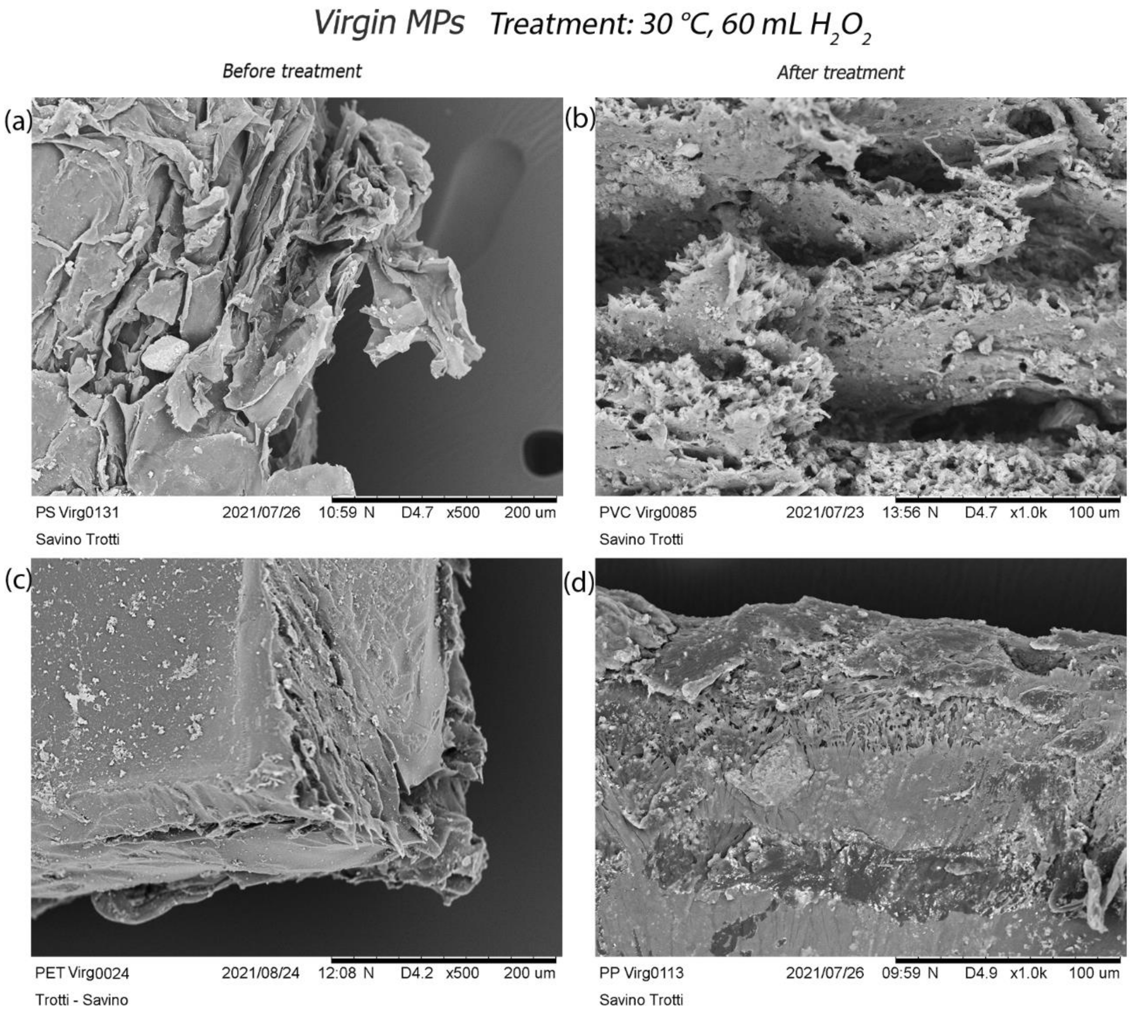

Figure S1. Detail on the effects of treatment at 30°C on polymers: a) slight damage to the PS; b) formation of small holes on the surface of the PVC; c) corrosion of PET margins; d) abrasion and corrosion of PP margins.

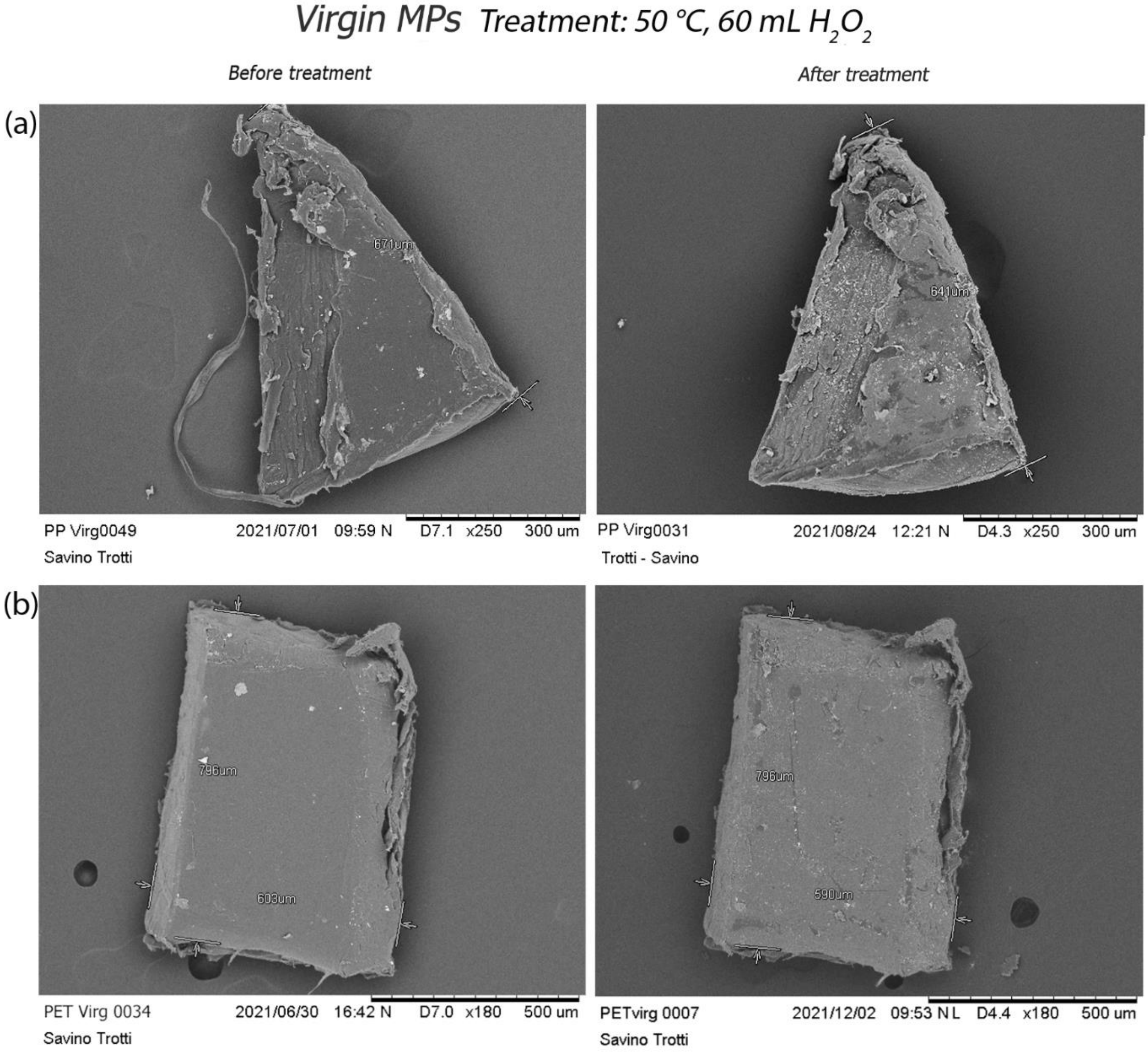

Figure S2. Slight size alteration caused by treatment at 50°C on virgin PP (a) and PET (b).

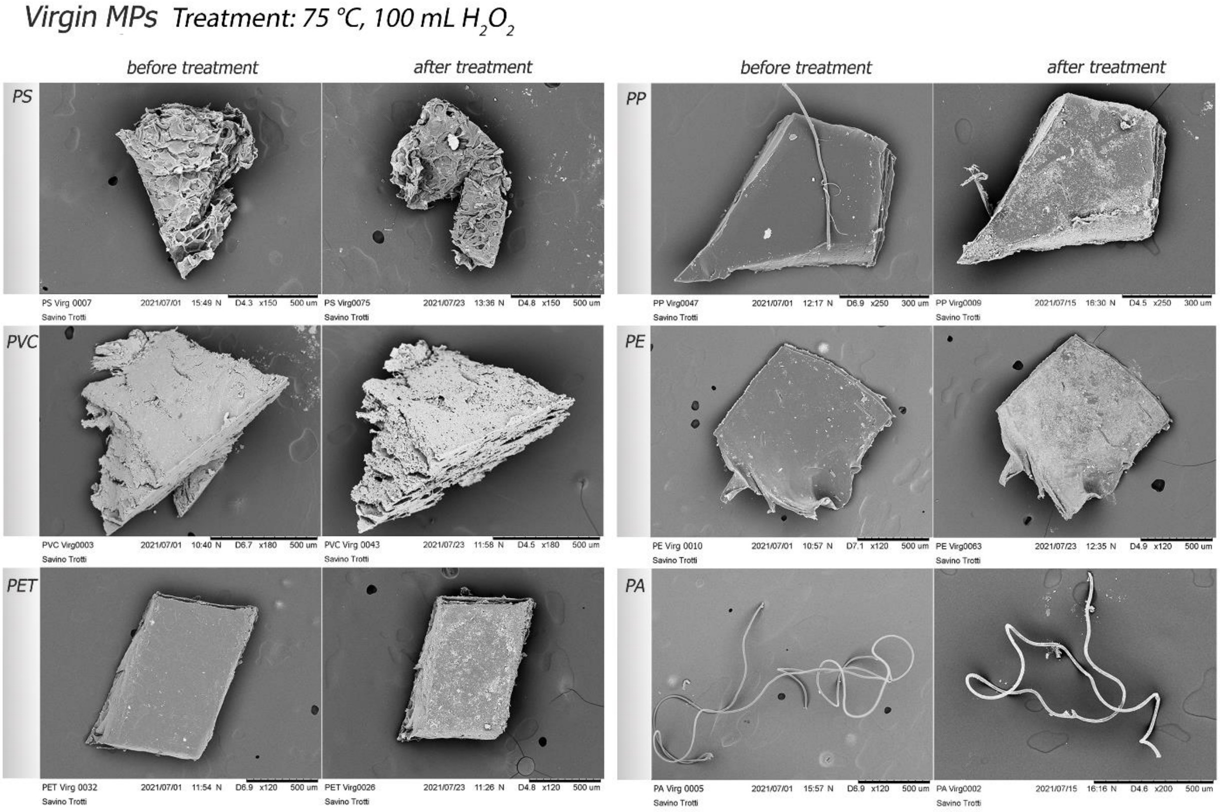

Figure S3. Overview of virgin polymer morphology before and after treatment at 75°C: loss of polymer material in PS; formation of large holes in the inside of PVC; corrosion of PP and PET margins, high resistance to PE and PA treatment.

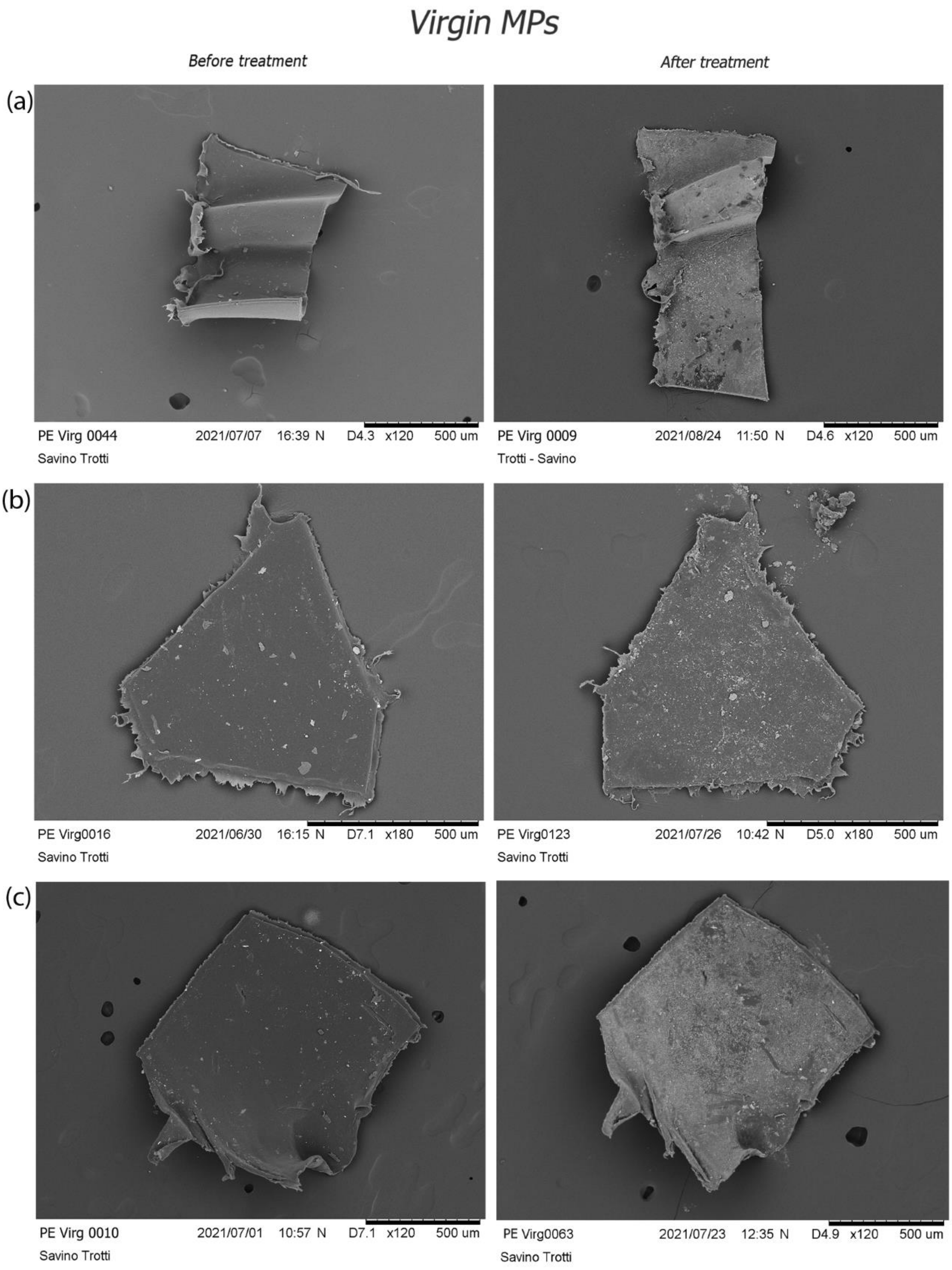

Figure S4. Visual demonstration of the high resistance of virgin PE after tratment at 30°C (a); treatment at 50°C (b), and 75°C (c).

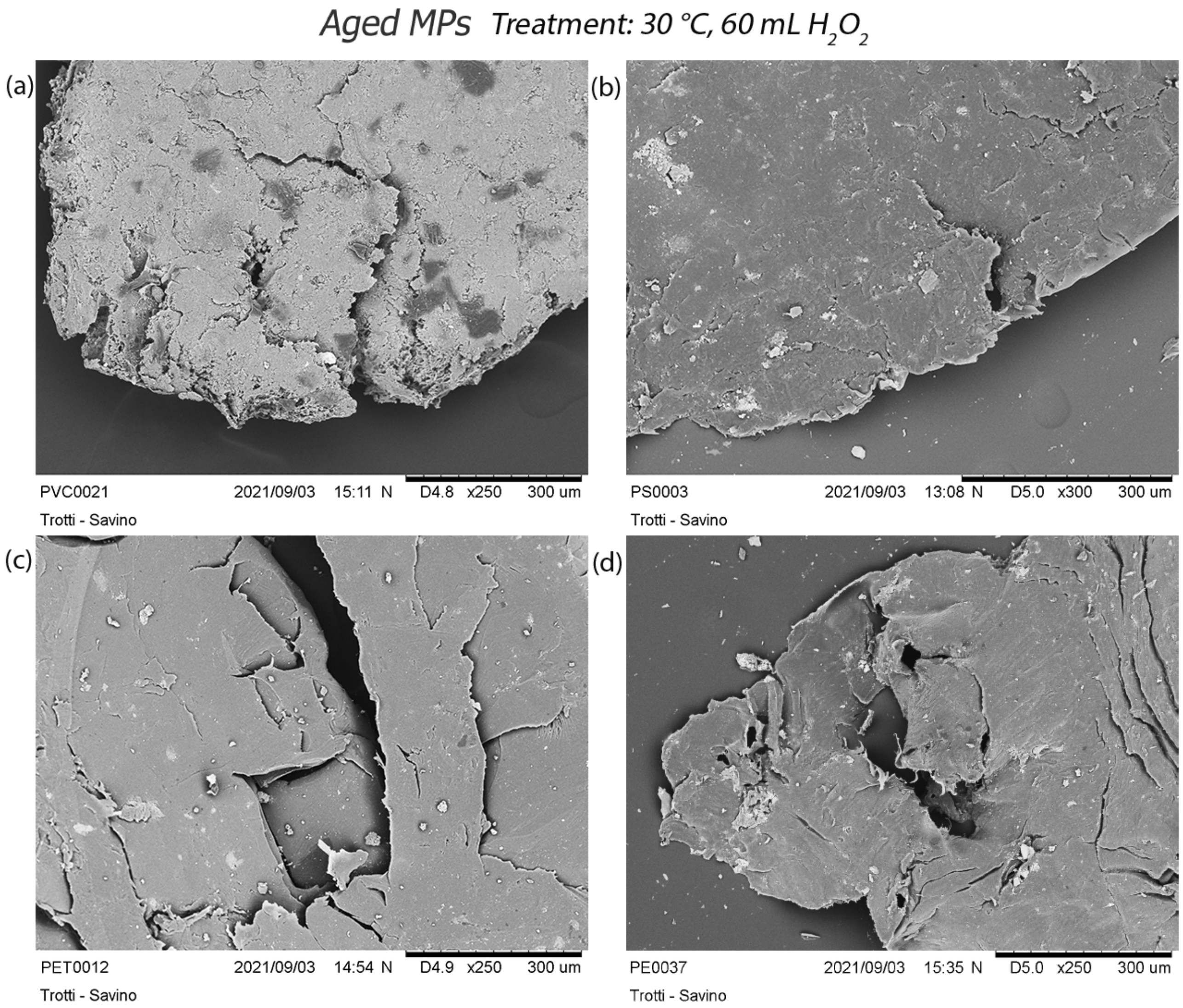

Figure S5. Focus on damage caused by treatment at 30°C on aged MPs: formation of cracks and breaks on PVC (a); PS (b); PET (c) and PE (d).

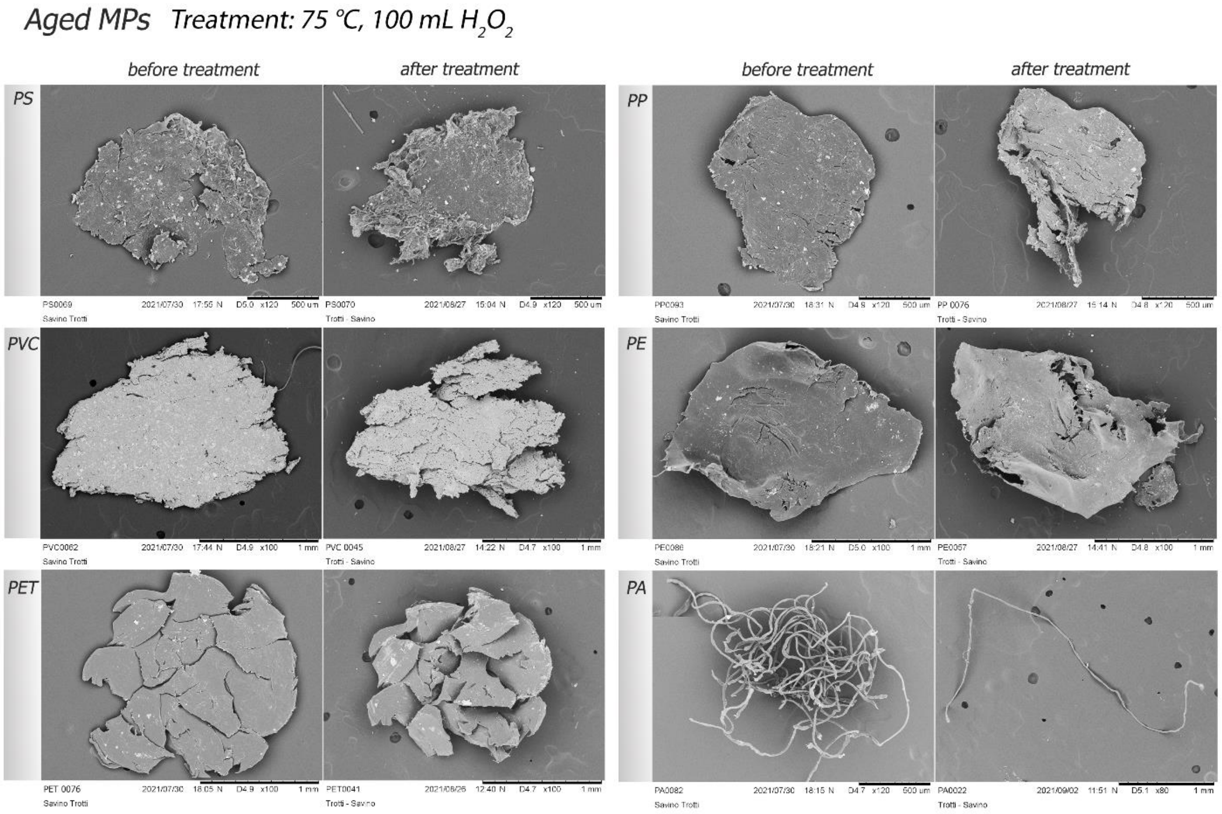

Figure S6. Overview of aged polymer morphology before and after treatment at 75°C: loss of material of PVC, PS and PP particles, cracks expansion of PET, PE corrosion and a fraying of PA.
